# 4,4′-(Anthracene-9,10-di­yl)dibenzoic acid dimethyl­formamide disolvate

**DOI:** 10.1107/S1600536809014858

**Published:** 2009-05-07

**Authors:** Hong Li, Zhi-Qiang Wang, Liu-Zhi Yang, Yan-Qi Liu, Duo-Bin Mao

**Affiliations:** a School of Food and Biological Engineering, Zhengzhou University of Light Industry, Zhengzhou 450002, People’s Republic of China; b College of Chemistry and Chemical Engineering, Luoyang Normal University, Luoyang 471022, People’s Republic of China

## Abstract

In the title compound, C_28_H_18_O_4_·2C_3_H_7_NO, the dihedral angle between the benzene rings and the anthracene system is 74.05 (12)°. A crystallographic inversion centre is located in the middle of the anthracene unit. The dimethyl­formamide solvent mol­ecules are partially disordered over two positions of approximately equal occupancy [0.529 (6):0.471 (6)]. Inter­molecular O—H⋯O hydrogen bonds with the major occupancy formamide O atom as acceptor result in the formation of 2:1 solvate–complex aggregates, which are alternately linked to shorter solvate units *via* weak inter­molecular C—H⋯O contacts generated from the rotational disorder of the formamide O atom (minor occupancy component). Weak C—H⋯π inter­actions between the solvent mol­ecules as the donor and the outer anthracene rings support these contacts in the crystal structure for both disorder components.

## Related literature

For the structure of 4-(2,5-dihexyl­oxyphen­yl)benzoic acid and the syntheses of related compounds, see: Li *et al.* (2008 ▶). For palladium-catalysed Suzuki coupling reactions, see: Xu *et al.* (2006 ▶, 2008 ▶); Li *et al.* (2006 ▶) and literature cited therein.
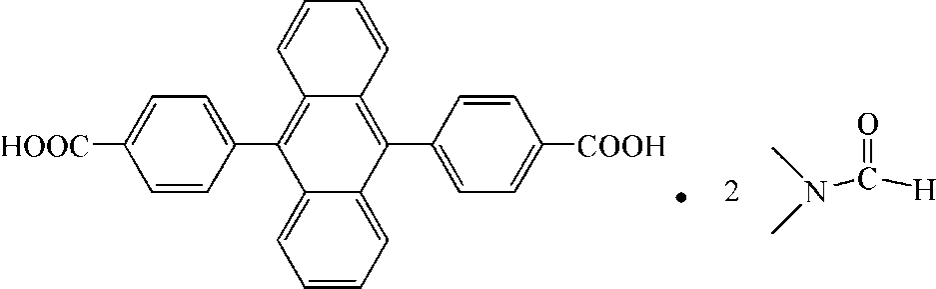


## Experimental

### 

#### Crystal data

C_28_H_18_O_4_·2C_3_H_7_NO
*M* *_r_* = 564.62
Triclinic, 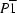

*a* = 7.3692 (15) Å
*b* = 8.9981 (18) Å
*c* = 12.124 (2) Å
α = 71.157 (3)°
β = 77.640 (3)°
γ = 79.754 (3)°
*V* = 738.0 (3) Å^3^
*Z* = 1
Mo *K*α radiation
μ = 0.09 mm^−1^
*T* = 295 K
0.23 × 0.16 × 0.06 mm

#### Data collection

Bruker SMART APEX CCD area-detector diffractometer
Absorption correction: multi-scan (*SADABS*; Sheldrick, 1996 ▶) *T*_min_ = 0.980, *T*_max_ = 0.994
5691 measured reflections
2721 independent reflections
1467 reflections with *I* > 2σ(*I*)
*R*_int_ = 0.028

#### Refinement

*R*[*F* ^2^ > 2σ(*F* ^2^)] = 0.053
*wR*(*F* ^2^) = 0.162
*S* = 1.02
2721 reflections
203 parameters
H-atom parameters constrained
Δρ_max_ = 0.16 e Å^−3^
Δρ_min_ = −0.20 e Å^−3^

### 

Data collection: *APEX2* (Bruker, 2004 ▶); cell refinement: *SAINT* (Bruker, 2004 ▶); data reduction: *SAINT*; program(s) used to solve structure: *SHELXS97* (Sheldrick, 2008 ▶); program(s) used to refine structure: *SHELXL97* (Sheldrick, 2008 ▶); molecular graphics: *SHELXTL* (Sheldrick, 2008 ▶); software used to prepare material for publication: *SHELXTL* and *PLATON* (Spek, 2009 ▶).

## Supplementary Material

Crystal structure: contains datablocks global, I. DOI: 10.1107/S1600536809014858/si2166sup1.cif

Structure factors: contains datablocks I. DOI: 10.1107/S1600536809014858/si2166Isup2.hkl

Additional supplementary materials:  crystallographic information; 3D view; checkCIF report

## Figures and Tables

**Table 1:** Hydrogen-bond geometry (Å, °)

*D*—H⋯*A*	*D*—H	H⋯*A*	*D*⋯*A*	*D*—H⋯*A*
O2—H2*D*⋯O3^i^	0.82	1.79	2.603 (4)	170
C5—H5⋯O3′	0.93	2.63	3.478 (5)	152
C16—H16*A*⋯*Cg*1	0.96	2.91	3.485 (3)	120

Symmetry code: (i) 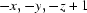. *Cg*1 is the centroid of the anthracene ring C8,C9,C10,C12*A*–C14*A*.

**Fig. 1.**
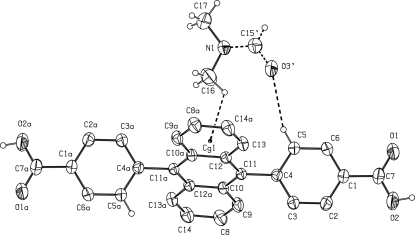
The molecular structure of the title compound with displacement ellipsoids at the 30% probability level (suffix A denotes the symmetry code: -x + 1, -y + 1, -z). Weak C—H···π and C—H···O hydrogen bonding contacts are indicated with dashed lines. Cg1 is the centroid of the anthracene ring C8, C9, C10, C12A, C13A, C14A.

**Fig. 2.**
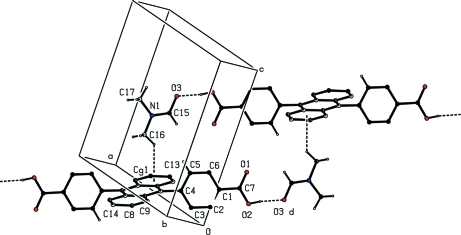
Partial view of the crystal packing showing the intermolecular O—H···O hydrogen bonds and weak C—H···π interactions. Cg1 is the centroid of the anthracene ring (C8, C9, C10, C12A, C13A, C14A).

## supplementary crystallographic information

### Comment

Cyclopalladated ferrocenylimine complexs with monophosphino ligands were successfully used as catalysts for Suzuki reactions (Xu *et al.* 2006; Li *et al.*, 2006; Xu *et al.* 2008). We have recently reported that the structure of 4-(2,5-dihexyloxyphenyl)benzoic acid was obtained from the Suzuki coupling reaction (Li *et al.*, 2008). The title compound was derived from the Suzuki reaction of 9,10-dibromoanthracene and 4-carboxyphenylboronic acid.

In the title compound (Fig.1), the dihedral angle between benzene rings and anthracene rings is 74.05 (12)°. A crystallographic inversion centre is in the middle of the anthracene unit, and an approximate two-fold pseudo rotation axis is running along the plane of the anthracene unit. The dimethylformamide solvent molecules are partially disordered over two positions, O3 and O3', of approximately equal occupancy, (0.529 (6) and 0.471 (6), respectively. The different intermolecular hydrogen bonding contacts are shown in Fig. 1 (O3' is the acceptor) and Fig. 2 (with O3 as acceptor). The intermolecular O—H···O hydrogen bonds result in the formation of long 2:1 solvate:complex aggregates, (Table 1) which are alternately linked *via* weak intermolecular C—H···O contacts generated from the rotational disorder of the formamide oxygen atom (0.471 (6) site occupancy). C—H···π interactions support these contacts in the crystal structure foming a one-dimensional supramolecular architecture (Fig. 1 and Fig. 2).

### Experimental

The title compound was obtained from the Suzuki coupling reaction of 9,10-dibromoanthracene and 4-carboxyphenylboronic acid as described in the literature (Li *et al.*, 2008) and recrystallized from dimethylformamide at room temperature to give the desired crystals suitable for single-crystal X-ray diffraction.

### Refinement

H atoms attached to C atoms of the title compound were placed in geometrically idealized positions and treated as riding with C—H distances constrained to 0.93 (aromatic CH), or 0.96 Å (methyl CH_3_), and constrained to ride on their parent atoms, with *U*_iso_(H) = 1.2*U*_eq_(C) (1.5*U*_eq_ for methyl H). The atom-site occupancies for the rotational disordered formamide oxygen atoms O3 and O3' refined to a ratio of 0.53/0.47.

Alert levels A and B for short intermolecular O1···O3' and H2D···H15' contacts with distances of2.50 Å and 2.01 Å may be explained by the difficulties to split the whole solvent molecule due to the pseudo two-fold rotation of the methyl groups around the N1—C15 axis. BUMP instruction or splitting of the whole solvent molecule resulted in unstable refinements. Introduction of shift-limiting restraints (DAMP instruction) resulted in larger R-values without improving the geometries. Therefore the partial disorder refinement (O3, O3', H15, H15') was preferred as a compromise.

### Crystal data

**Table:**

C_28_H_18_O_4_·2C_3_H_7_NO	*Z* = 1
*M**_r_* = 564.62	*F*(000) = 298
Triclinic, *P*1	*D*_x_ = 1.270 Mg m^−^^3^
Hall symbol: -P 1	Mo *K*α radiation, λ = 0.71073 Å
*a* = 7.3692 (15) Å	Cell parameters from 879 reflections
*b* = 8.9981 (18) Å	θ = 2.9–22.0°
*c* = 12.124 (2) Å	µ = 0.09 mm^−^^1^
α = 71.157 (3)°	*T* = 295 K
β = 77.640 (3)°	Block, colourless
γ = 79.754 (3)°	0.23 × 0.16 × 0.06 mm
*V* = 738.0 (3) Å^3^	

### Data collection

**Table:**

Bruker SMART APEX CCD area-detector diffractometer	2721 independent reflections
Radiation source: fine-focus sealed tube	1467 reflections with *I* > 2σ(*I*)
graphite	*R*_int_ = 0.028
φ and ω scans	θ_max_ = 25.5°, θ_min_ = 2.4°
Absorption correction: multi-scan (*SADABS*; Sheldrick, 1996)	*h* = −8→8
*T*_min_ = 0.980, *T*_max_ = 0.994	*k* = −10→9
5691 measured reflections	*l* = −14→14

### Refinement

**Table:**

Refinement on *F*^2^	Primary atom site location: structure-invariant direct methods
Least-squares matrix: full	Secondary atom site location: difference Fourier map
*R*[*F*^2^ > 2σ(*F*^2^)] = 0.053	Hydrogen site location: inferred from neighbouring sites
*wR*(*F*^2^) = 0.162	H-atom parameters constrained
*S* = 1.02	*w* = 1/[σ^2^(*F*_o_^2^) + (0.0732*P*)^2^ + 0.04*P*] where *P* = (*F*_o_^2^ + 2*F*_c_^2^)/3
2721 reflections	(Δ/σ)_max_ < 0.001
203 parameters	Δρ_max_ = 0.16 e Å^−^^3^
0 restraints	Δρ_min_ = −0.20 e Å^−^^3^

### Special details

**Table:**

Geometry. All esds (except the esd in the dihedral angle between two l.s. planes) are estimated using the full covariance matrix. The cell esds are taken into account individually in the estimation of esds in distances, angles and torsion angles; correlations between esds in cell parameters are only used when they are defined by crystal symmetry. An approximate (isotropic) treatment of cell esds is used for estimating esds involving l.s. planes.
Refinement. Refinement of *F*^2^ against ALL reflections. The weighted *R*-factor *wR* and goodness of fit *S* are based on *F*^2^, conventional *R*-factors *R* are based on *F*, with *F* set to zero for negative *F*^2^. The threshold expression of *F*^2^ > σ(*F*^2^) is used only for calculating *R*-factors(gt) *etc*. and is not relevant to the choice of reflections for refinement. *R*-factors based on *F*^2^ are statistically about twice as large as those based on *F*, and *R*- factors based on ALL data will be even larger.

### Fractional atomic coordinates and isotropic or equivalent isotropic displacement parameters (Å^2^)

**Table:**

	*x*	*y*	*z*	*U*_iso_*/*U*_eq_	Occ. (<1)
C15	0.5707 (5)	0.2790 (4)	0.4860 (3)	0.0742 (9)	0.529 (6)
H15	0.4771	0.2595	0.4536	0.089*	0.529 (6)
O3	0.5840 (5)	0.2039 (5)	0.5938 (4)	0.0918 (18)	0.529 (6)
C15'	0.5707 (5)	0.2790 (4)	0.4860 (3)	0.0742 (9)	0.471 (6)
H15'	0.5944	0.2141	0.5598	0.089*	0.471 (6)
O3'	0.4309 (7)	0.2700 (6)	0.4479 (4)	0.096 (2)	0.471 (6)
N1	0.6843 (3)	0.3826 (3)	0.41928 (19)	0.0637 (6)	
C16	0.6620 (5)	0.4705 (4)	0.2994 (3)	0.0999 (12)	
H16A	0.5498	0.4476	0.2831	0.150*	
H16B	0.7677	0.4407	0.2459	0.150*	
H16C	0.6535	0.5816	0.2895	0.150*	
C17	0.8377 (4)	0.4104 (5)	0.4627 (3)	0.0945 (11)	
H17A	0.8375	0.3450	0.5429	0.142*	
H17B	0.8249	0.5196	0.4598	0.142*	
H17C	0.9532	0.3852	0.4146	0.142*	
O1	−0.1924 (3)	−0.0937 (3)	0.42504 (19)	0.1024 (9)	
O2	−0.3377 (3)	−0.0181 (3)	0.2730 (2)	0.1090 (9)	
H2D	−0.4140	−0.0728	0.3213	0.163*	
C1	−0.0616 (3)	0.0915 (3)	0.2520 (2)	0.0496 (6)	
C2	−0.0651 (4)	0.1757 (3)	0.1348 (2)	0.0638 (8)	
H2	−0.1589	0.1662	0.0978	0.077*	
C3	0.0713 (4)	0.2746 (3)	0.0719 (2)	0.0594 (7)	
H3	0.0669	0.3313	−0.0068	0.071*	
C4	0.2133 (3)	0.2901 (3)	0.1243 (2)	0.0445 (6)	
C5	0.2169 (3)	0.2037 (3)	0.2414 (2)	0.0525 (7)	
H5	0.3117	0.2117	0.2783	0.063*	
C6	0.0805 (3)	0.1053 (3)	0.3045 (2)	0.0537 (7)	
H6	0.0852	0.0480	0.3831	0.064*	
C7	−0.2053 (4)	−0.0146 (3)	0.3219 (3)	0.0643 (8)	
C8	0.6573 (4)	0.1754 (3)	−0.1369 (2)	0.0640 (8)	
H8	0.6611	0.0821	−0.1552	0.077*	
C9	0.5159 (4)	0.2148 (3)	−0.0568 (2)	0.0549 (7)	
H9	0.4239	0.1477	−0.0204	0.066*	
C10	0.5040 (3)	0.3575 (3)	−0.02629 (19)	0.0432 (6)	
C11	0.3592 (3)	0.3985 (3)	0.05850 (19)	0.0419 (6)	
C12	0.3533 (3)	0.5398 (3)	0.08512 (19)	0.0419 (6)	
C13	0.2061 (3)	0.5896 (3)	0.1681 (2)	0.0514 (7)	
H13	0.1110	0.5259	0.2059	0.062*	
C14	0.2008 (4)	0.7263 (3)	0.1932 (2)	0.0620 (8)	
H14	0.1031	0.7554	0.2478	0.074*	

### Atomic displacement parameters (Å^2^)

**Table:**

	*U*^11^	*U*^22^	*U*^33^	*U*^12^	*U*^13^	*U*^23^
C15	0.066 (2)	0.089 (2)	0.066 (2)	−0.0197 (18)	−0.0072 (17)	−0.0164 (19)
O3	0.082 (3)	0.114 (4)	0.071 (3)	−0.054 (3)	−0.012 (2)	0.006 (3)
C15'	0.066 (2)	0.089 (2)	0.066 (2)	−0.0197 (18)	−0.0072 (17)	−0.0164 (19)
O3'	0.087 (4)	0.120 (4)	0.079 (3)	−0.060 (3)	−0.015 (3)	0.000 (3)
N1	0.0574 (14)	0.0753 (17)	0.0547 (14)	−0.0202 (12)	−0.0040 (11)	−0.0113 (12)
C16	0.088 (2)	0.119 (3)	0.071 (2)	−0.022 (2)	−0.0099 (18)	0.004 (2)
C17	0.079 (2)	0.125 (3)	0.086 (2)	−0.045 (2)	−0.0063 (19)	−0.028 (2)
O1	0.0933 (16)	0.128 (2)	0.0722 (15)	−0.0639 (15)	−0.0176 (13)	0.0188 (14)
O2	0.0826 (16)	0.137 (2)	0.0938 (17)	−0.0672 (15)	−0.0245 (14)	0.0188 (15)
C1	0.0454 (14)	0.0470 (15)	0.0548 (16)	−0.0134 (12)	−0.0041 (12)	−0.0114 (12)
C2	0.0535 (16)	0.0701 (19)	0.0652 (18)	−0.0227 (14)	−0.0159 (14)	−0.0045 (15)
C3	0.0605 (16)	0.0624 (18)	0.0512 (15)	−0.0233 (14)	−0.0133 (13)	−0.0001 (13)
C4	0.0460 (14)	0.0408 (14)	0.0468 (14)	−0.0098 (11)	−0.0061 (11)	−0.0115 (12)
C5	0.0541 (15)	0.0553 (16)	0.0474 (15)	−0.0189 (13)	−0.0085 (12)	−0.0077 (13)
C6	0.0593 (16)	0.0519 (16)	0.0453 (14)	−0.0170 (13)	−0.0041 (12)	−0.0058 (12)
C7	0.0506 (17)	0.0634 (19)	0.074 (2)	−0.0191 (14)	−0.0030 (15)	−0.0121 (16)
C8	0.085 (2)	0.0442 (16)	0.0605 (17)	−0.0144 (14)	0.0043 (15)	−0.0192 (14)
C9	0.0667 (17)	0.0400 (15)	0.0549 (16)	−0.0186 (12)	0.0012 (13)	−0.0107 (12)
C10	0.0494 (14)	0.0359 (14)	0.0416 (13)	−0.0110 (11)	−0.0061 (11)	−0.0054 (11)
C11	0.0453 (13)	0.0386 (14)	0.0391 (13)	−0.0119 (10)	−0.0083 (11)	−0.0034 (11)
C12	0.0426 (13)	0.0404 (14)	0.0404 (13)	−0.0080 (10)	−0.0063 (10)	−0.0074 (11)
C13	0.0485 (14)	0.0494 (16)	0.0499 (15)	−0.0125 (12)	0.0041 (12)	−0.0104 (12)
C14	0.0724 (18)	0.0492 (17)	0.0573 (17)	−0.0080 (14)	0.0079 (14)	−0.0178 (14)

### Geometric parameters (Å, °)

**Table:**

C15—O3	1.279 (4)	C3—H3	0.9300
C15—N1	1.314 (4)	C4—C5	1.385 (3)
C15—H15	0.9300	C4—C11	1.499 (3)
N1—C17	1.434 (4)	C5—C6	1.389 (3)
N1—C16	1.441 (3)	C5—H5	0.9300
C16—H16A	0.9600	C6—H6	0.9300
C16—H16B	0.9600	C8—C9	1.346 (3)
C16—H16C	0.9600	C8—C14^i^	1.406 (4)
C17—H17A	0.9600	C8—H8	0.9300
C17—H17B	0.9600	C9—C10	1.430 (3)
C17—H17C	0.9600	C9—H9	0.9300
O1—C7	1.238 (3)	C10—C11	1.403 (3)
O2—C7	1.255 (3)	C10—C12^i^	1.438 (3)
O2—H2D	0.8200	C11—C12	1.401 (3)
C1—C6	1.378 (3)	C12—C13	1.428 (3)
C1—C2	1.381 (3)	C12—C10^i^	1.438 (3)
C1—C7	1.485 (3)	C13—C14	1.353 (3)
C2—C3	1.392 (3)	C13—H13	0.9300
C2—H2	0.9300	C14—C8^i^	1.406 (4)
C3—C4	1.382 (3)	C14—H14	0.9300
			
O3—C15—N1	122.5 (4)	C4—C5—C6	120.7 (2)
O3—C15—H15	118.8	C4—C5—H5	119.6
N1—C15—H15	118.8	C6—C5—H5	119.6
C15—N1—C17	121.0 (3)	C1—C6—C5	120.8 (2)
C15—N1—C16	121.5 (3)	C1—C6—H6	119.6
C17—N1—C16	117.5 (2)	C5—C6—H6	119.6
N1—C16—H16A	109.5	O1—C7—O2	122.7 (3)
N1—C16—H16B	109.5	O1—C7—C1	119.7 (3)
H16A—C16—H16B	109.5	O2—C7—C1	117.6 (3)
N1—C16—H16C	109.5	C9—C8—C14^i^	120.8 (3)
H16A—C16—H16C	109.5	C9—C8—H8	119.6
H16B—C16—H16C	109.5	C14^i^—C8—H8	119.6
N1—C17—H17A	109.5	C8—C9—C10	121.5 (2)
N1—C17—H17B	109.5	C8—C9—H9	119.2
H17A—C17—H17B	109.5	C10—C9—H9	119.2
N1—C17—H17C	109.5	C11—C10—C9	122.0 (2)
H17A—C17—H17C	109.5	C11—C10—C12^i^	119.9 (2)
H17B—C17—H17C	109.5	C9—C10—C12^i^	118.1 (2)
C7—O2—H2D	109.5	C12—C11—C10	119.9 (2)
C6—C1—C2	118.9 (2)	C12—C11—C4	119.3 (2)
C6—C1—C7	119.4 (2)	C10—C11—C4	120.8 (2)
C2—C1—C7	121.6 (2)	C11—C12—C13	122.3 (2)
C1—C2—C3	120.2 (2)	C11—C12—C10^i^	120.2 (2)
C1—C2—H2	119.9	C13—C12—C10^i^	117.5 (2)
C3—C2—H2	119.9	C14—C13—C12	122.0 (2)
C4—C3—C2	121.2 (2)	C14—C13—H13	119.0
C4—C3—H3	119.4	C12—C13—H13	119.0
C2—C3—H3	119.4	C13—C14—C8^i^	120.0 (2)
C3—C4—C5	118.1 (2)	C13—C14—H14	120.0
C3—C4—C11	121.9 (2)	C8^i^—C14—H14	120.0
C5—C4—C11	119.9 (2)		
			
O3—C15—N1—C17	3.6 (5)	C8—C9—C10—C11	−178.9 (2)
O3—C15—N1—C16	−177.4 (4)	C8—C9—C10—C12^i^	0.9 (4)
C6—C1—C2—C3	−1.1 (4)	C9—C10—C11—C12	−179.8 (2)
C7—C1—C2—C3	179.7 (2)	C12^i^—C10—C11—C12	0.4 (4)
C1—C2—C3—C4	0.5 (4)	C9—C10—C11—C4	2.2 (3)
C2—C3—C4—C5	0.3 (4)	C12^i^—C10—C11—C4	−177.6 (2)
C2—C3—C4—C11	−178.8 (2)	C3—C4—C11—C12	106.8 (3)
C3—C4—C5—C6	−0.5 (4)	C5—C4—C11—C12	−72.3 (3)
C11—C4—C5—C6	178.6 (2)	C3—C4—C11—C10	−75.2 (3)
C2—C1—C6—C5	0.9 (4)	C5—C4—C11—C10	105.7 (3)
C7—C1—C6—C5	−179.9 (2)	C10—C11—C12—C13	178.1 (2)
C4—C5—C6—C1	−0.1 (4)	C4—C11—C12—C13	−3.9 (3)
C6—C1—C7—O1	−2.6 (4)	C10—C11—C12—C10^i^	−0.4 (4)
C2—C1—C7—O1	176.6 (3)	C4—C11—C12—C10^i^	177.6 (2)
C6—C1—C7—O2	176.5 (3)	C11—C12—C13—C14	−179.8 (2)
C2—C1—C7—O2	−4.3 (4)	C10^i^—C12—C13—C14	−1.3 (4)
C14^i^—C8—C9—C10	0.3 (4)	C12—C13—C14—C8^i^	0.1 (4)

Symmetry codes: (i) −*x*+1, −*y*+1, −*z*.

### Hydrogen-bond geometry (Å, °)

**Table:**

*D*—H···*A*	*D*—H	H···*A*	*D*···*A*	*D*—H···*A*
O2—H2D···O3^ii^	0.82	1.79	2.603 (4)	170
C5—H5···O3'	0.93	2.63	3.478 (5)	152
C16—H16A···Cg1	0.96	2.91	3.485 (3)	120

Symmetry codes: (ii) −*x*, −*y*, −*z*+1.
